# Severe acute respiratory failure due to a multifactorial diffuse alveolar haemorrhage

**DOI:** 10.1002/rcr2.531

**Published:** 2020-03-08

**Authors:** Ernesto Crisafulli, Barbara Burgazzi, Maria Majori, Walter Serra, Alfredo Chetta

**Affiliations:** ^1^ Department of Medicine and Surgery, Respiratory Disease and Lung Function Unit University of Parma Parma Italy; ^2^ Department of Medicine, Respiratory Medicine Unit and Section of Internal Medicine University of Verona and Azienda Ospedaliera Universitaria Integrata of Verona Verona Italy; ^3^ Department of Internal Medicine, Interventional Pneumology Unit University Hospital of Parma Parma Italy; ^4^ Cardiology Division, Surgery Department University Hospital of Parma Parma Italy

**Keywords:** Acute respiratory failure, clopidogrel, diffuse alveolar haemorrhage, warfarin

## Abstract

Diffuse alveolar haemorrhage (DAH) is a life‐threatening syndrome caused by infection, coagulation disorders or autoimmune diseases. We here report the case of an 81‐year‐old male subject affected by a multifactorial DAH, in which the bleeding was related to the administration of clopidogrel and warfarin, both implicated in the context of a polycythaemia. He developed a severe acute respiratory failure treated with a ventilatory support by means of a continuous positive airway pressure (C‐PAP) therapy. An improvement of patient's clinical conditions was observed only after clopidogrel and warfarin discontinuation.

## Graphical abstract

We here report the case of an 81‐year‐old male subject affected by a multifactorial diffuse alveolar haemorrhage (DAH), in which the bleeding was related to the administration of clopidogrel and warfarin, both implicated in the context of a polycythaemia. He developed a severe acute respiratory failure treated with a ventilatory support by means of a continuous positive airway pressure (C‐PAP) therapy. An improvement of patient's clinical conditions was observed only after clopidogrel and warfarin discontinuation.

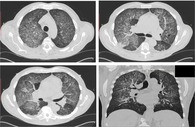

## Introduction

Diffuse alveolar haemorrhage (DAH) is a rare, life‐threatening disorder caused by the accumulation of red blood cells in the lung parenchyma, originating from the alveolar capillaries 1. This condition may be caused by infection, coagulation disorders, autoimmune diseases involving the lung, and use of drugs, such as oral anticoagulants or antiplatelet therapy 2. DAH is suspected based on clinical presentation and on laboratory and radiological findings; however, the diagnosis should be confirmed by bronchoscopy and bronchoalveolar lavage (BAL) 1.

## Case Report

**Figure 1:**
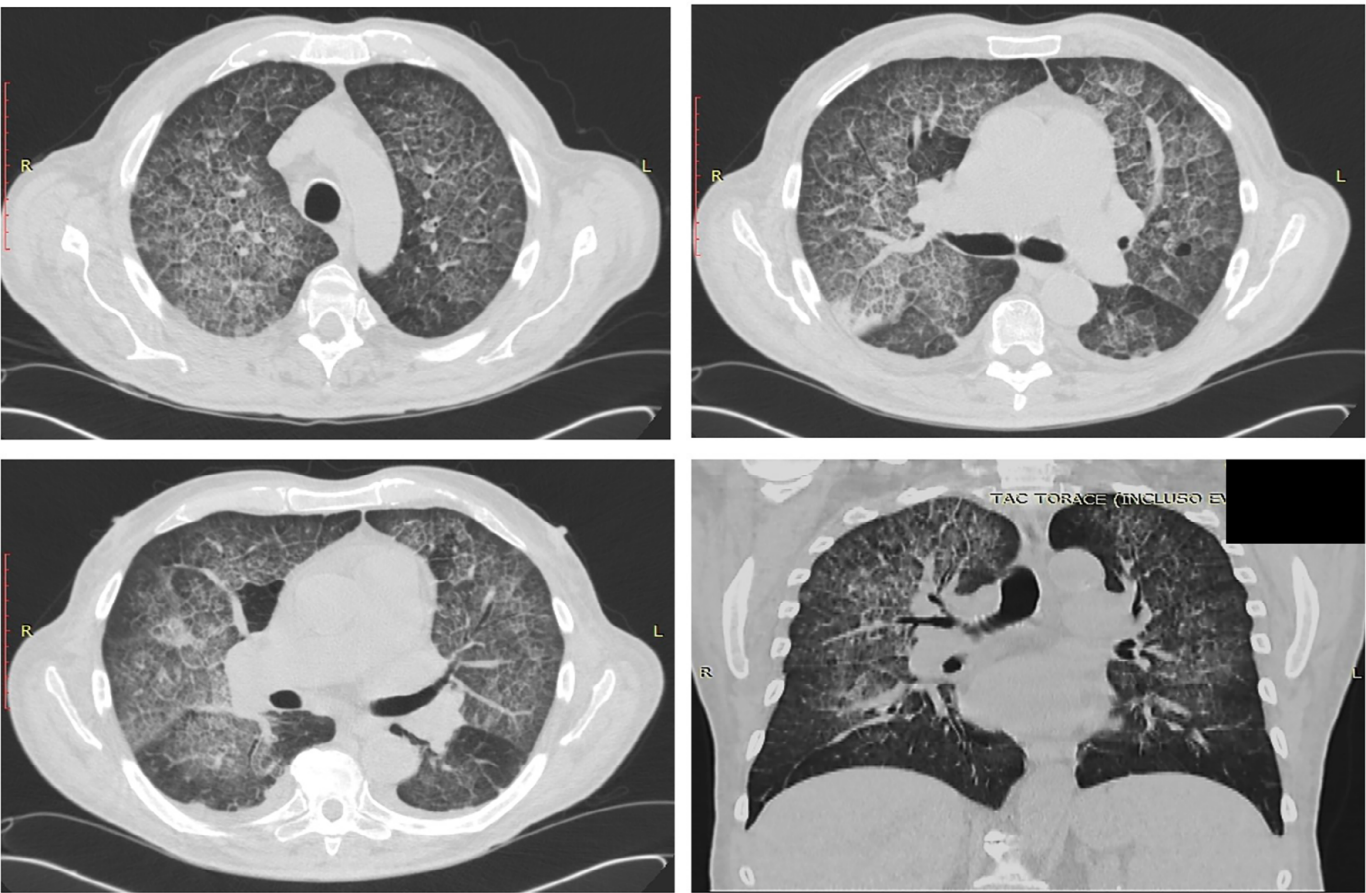
Chest computed tomography scan at admission to hospital.

In January 2019, an 81‐year‐old male subject, retired after working as auto mechanic, was transferred to our Respiratory Disease and Lung Function Unit for a severe acute respiratory failure (ARF), with a suspected acute interstitial lung disease. The patient had never smoked and denied any alcohol abuse. He reported a clinical history of polycythaemia vera (from the 2005), treated with bloodletting and, from the 2017, with hydroxyurea. In 2005, the patient had a myocardial infarction, treated with thrombolysis. In August 2018 he had a deep vein thrombosis of the right femoral vein, treated with edoxaban. On 18 December 2018, the patient was admitted to the cardiology unit of our hospital for a relapse of the myocardial infarction, treated with a drug‐eluting stent and a dual antiplatelet therapy (aspirin 100 mg/die and clopidogrel 75 mg/die). Due to an intercurrent acute renal failure, the therapy with edoxaban was replaced with warfarin. On 3 January 2019, he was admitted to the emergency department for a severe dyspnoea at rest. The arterial blood gas defined a severe hypoxemic ARF, with a ratio of partial pressure of arterial oxygen to the fraction of inspired oxygen (PaO_2_/FiO_2_) of 179. The serum analysis revealed macrocytic anaemia (haemoglobin‐Hb 10.3 g/dL), leucocitosis (leucocytes 26.1 × 10^9^/L) and an increased level of C‐reactive protein (47 mg/L). A chest computed tomography (CT) scan showed diffuse ground‐glass opacities with crazy‐paving pattern compatible with an acute interstitial disease (Fig. 1). He complained of haemoptysis and, for this reason, he stopped the warfarin therapy the same day.

**Figure 2:**
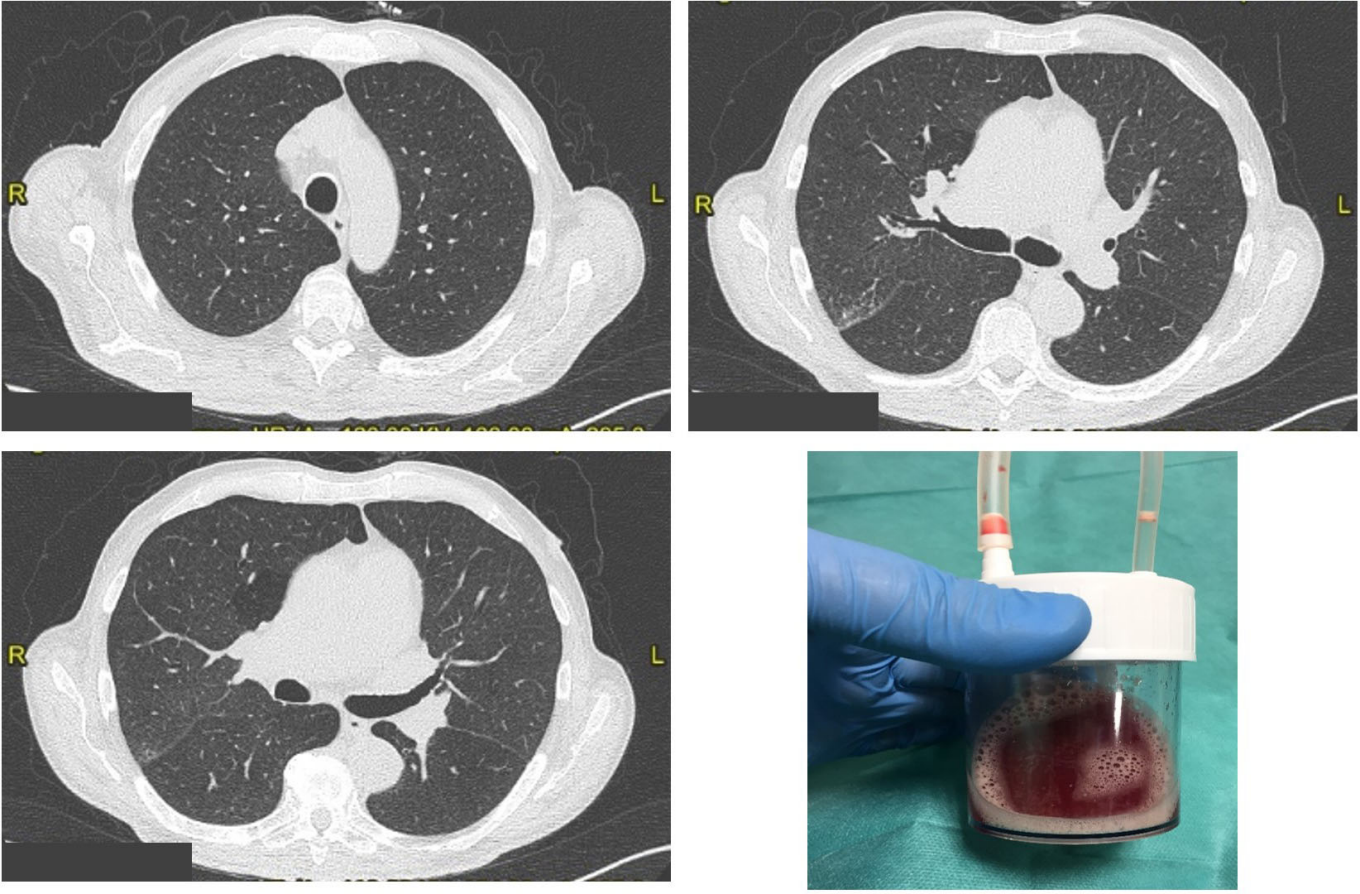
Chest computed tomography scan after resolution of diffuse alveolar haemorrhage and the bronchoalveolar lavage confirming the presence of blood.

On 11 January 2019, at admission to our unit, the patient had a severe respiratory distress with a worsening of the PaO_2_/FiO_2_ ratio (113). His values of red blood cells (RBC), haematocrit (Ht) and Hb were low (RBC 2.6 million cells/mm^3^, Ht 26% and Hb 7.6 g/dL, respectively). Consequently he was transfused. Microbiological urinary antigen tests against *Streptococcus pneumoniae* and *Legionella pneumophila* and serum antibodies against *S. pneumoniae* were negative. All autoantibodies for pulmonary vasculitis (antinuclear antibodies (ANA) and antineutrophil cytoplasmic autoantibodies (ANCA)) were negative as well. We started empirically an antibiotic therapy with trimethoprim‐sulfamethoxazole (1600/320 mg/die) and levofloxacin (500 mg/die), together with a high‐dose steroid therapy (methylprednisolone, 1 mg/kg/die). Due to the severe hypoxemia, the patient underwent a ventilatory support with a continuous positive airway pressure (C‐PAP) and with oxygen supplementation at 8 L/min. On 17 January 2019, the patient discontinued the treatment with clopidogrel and since 21 January 2019 the patient showed a progressive improvement of perceived dyspnoea and of the PaO_2_/FiO_2_ ratio (227). On 24 January 2019, the patient underwent a flexible bronchoscopy with BAL, confirming the presence of blood (Fig. 2, lower right panel), with blood clots on the bronchial wall of the two bronchial hemi‐systems. On 29 January 2019, the patient resolved completely the ARF (PaO_2_/FiO_2_ ratio: 304), with a significant decrease in the dyspnoea perception. A CT scan on 5 February 2019 (Fig. 2) showed a complete radiological resolution of the diffuse ground‐glass opacities.

## Discussion

DAH is a syndrome characterized by bleeding into the alveolar space due to a damage to the alveolar microcirculation 1. Common symptoms are cough, dyspnoea, fever, and haemoptysis, which can occur in one‐third of patients only 2. DAH can be suspected on the basis of the clinical presentation, the laboratory data, and the chest CT scan, although the final diagnosis is confirmed by bronchoscopy and BAL.

The causes of DAH may be various 1. Vasculitis occurs in 88% of the cases (in this context the Wegener granulomatosis is the most frequent, followed by the Goodpasture syndrome) 1. DAH may be rarely caused by drugs as well 1, 2. In our case, in a context of a chronic illness (polycythaemia) the use of warfarin and clopidogrel have increased the risk of bleeding. The diagnosis was based on clinical presentation and was confirmed by high‐resolution CT (HRCT) scan and BAL. The discontinuation of warfarin and clopidogrel led to an improvement in ARF and to a complete clinical and radiological resolution. With regards to the use of clopidogrel few cases have been reported in literature 3, 4, 5. In a single patient, clopidogrel‐induced DAH occurred after coronary stent placement 3, in two patients after acute coronary syndrome 4, 5; in these latter cases, DAH was fatal 4, 5.

### Disclosure statement

Appropriate written informed consent was obtained for publication of this case report and accompanying images.
